# Respiratory sinus arrhythmia moderates the interpersonal consequences of brooding rumination

**DOI:** 10.1177/02654075221122059

**Published:** 2022-08-19

**Authors:** Warren Caldwell, Sasha MacNeil, Carsten Wrosch, Jennifer J. McGrath, Thanh T. Dang-Vu, Alexandre J. S. Morin, Jean-Philippe Gouin

**Affiliations:** 1 Department of Psychology, 5618 Concordia University, Montreal, Canada; 2 Department of Health, Kinesiology and Applied Physiology, 390875 Concordia University, Montreal, Canada

**Keywords:** Brooding rumination, respiratory sinus arrhythmia, heart rate variability, interpersonal stress, negative interpersonal behaviors, social support

## Abstract

Brooding rumination is an intrapersonal emotion regulation strategy associated with negative interpersonal consequences. Resting respiratory sinus arrhythmia (RSA), a psychophysiological marker of self-regulatory capacity, may buffer the association between maladaptive emotion regulation and negative interpersonal behaviors. The current work examines the moderating effect of RSA on the association between brooding rumination and different negative interpersonal consequences. Across three convenience samples, individuals with lower RSA showed a stronger association between brooding rumination and more negative interpersonal behaviors as well as less perception of received instrumental social support (Study 1; *n* = 154), higher levels of interviewer-rated interpersonal stress (Study 2; *n* = 42) and a stronger indirect association between brooding rumination and depressive symptoms via daily interpersonal stress (Study 3; *n* = 222). These findings highlight the negative interpersonal consequences of brooding rumination, particularly among individuals with lower RSA.

Brooding rumination is an intrapersonal emotion regulation strategy involving repetitive, passive, self-focused cognition about the causes and consequences of one’s experience of distress (Nolen-Hoeksema et al., 2008). This emotion regulation strategy has been associated with increased risk for dysphoric mood, negative interpersonal behaviors and reduced social support availability (Nolen-Hoeksema et al., 2008). However, the association between brooding rumination and negative interpersonal experiences has not been found in all studies, garnering interest in identifying moderators of these associations (Liu & Alloy, 2010). Respiratory sinus arrhythmia (RSA; Berntson et al., 1997), the fluctuations in time intervals between consecutive heartbeats linked to the respiration cycle, is a biomarker that is theorized to index a neurophysiological circuit that supports effective self-regulation in interpersonal relationships (Porges, 2003; Thayer & Lane, 2009). As such, greater RSA may mitigate the negative interpersonal consequences of rumination. The current set of studies examined whether RSA moderates the associations between brooding rumination and negative interpersonal behaviors and received social support (Study 1), chronic interpersonal stress (Study 2), and daily stressful interpersonal experiences (Study 3).

## Rumination, negative interpersonal behaviors and interpersonal stress

Emotion regulation is increasingly conceptualized as an interpersonal process, where attempts to influence the timing, experience and expression of emotion involves exchanges between intrapersonal regulation strategies and reactions from the social environment (Marroquín, 2011; Zaki & Craig Williams, 2013). Indeed, supportive social contexts are associated with more adaptive and effective intrapersonal emotion regulation (DeLongis & Holtzman, 2005; Holtzman et al., 2004), whereas social isolation may impair intrapersonal emotion regulation (Cacioppo & Hawkley, 2009). Reciprocally, the use of particular intrapersonal emotion regulation strategies are also associated with the quality of social interactions (Butler et al., 2003; Richards et al., 2003).

Nolen-Hoeksema et al. (2008) proposes brooding rumination is an intrapersonal emotion regulation strategy that enhances negative thinking, impairs problem-solving and interferes with instrumental behaviors. These *intra*personal difficulties may spill-over into *inter*personal exchanges because of persistent urges to discuss upsetting material and the perception of insufficient social support, leading to more negative social interactions, more negative social evaluations of the ruminating individual, and the erosion of social support resources over time (Nolen-Hoeksema et al., 2008). Empirically, brooding rumination increases the risk for various negative interpersonal behaviors, including excessive reassurance seeking (Potthoff et al., 1995; Stroud et al., 2018; Weinstock & Whisman, 2007), poor interpersonal problem-solving (Lyubomirsky & Nolen-Hoeksema, 1995), greater motivation to continue arguments (Carr et al., 2012) and aggression following interpersonal transgressions (Collins & Bell, 1997; Watkins et al., 2015). Rumination is also associated with lower satisfaction with social support (Nolen-Hoeksema & Davis, 1999) and greater daily withdrawal from romantic partners (King & DeLongis, 2014). Thus, brooding rumination is an intrapersonal emotion regulation strategy that increases risk for negative interpersonal behaviours.

Converging evidence shows brooding rumination is also associated with the development of interpersonal stress, both cross-sectionally (Lam et al., 2003) and longitudinally (McLaughlin & Nolen-Hoeksema, 2012; Stroud et al., 2015; 2018). Further, brooding rumination has been associated with poorer relationship quality, including the perception of less social support (Nolen-Hoeksema & Davis, 1999), more relationship conflict (Caldwell et al., 2019; King & DeLongis, 2014) and prospective decreases in satisfaction with personal, sexual and social relationships (Pearson, Watkins, Kuyken, & Mullan, 2010). However, not all studies have confirmed associations between brooding rumination and interpersonal stress (Hamilton et al., 2013, 2017; Shapero et al., 2013), or indeed with maladaptive outcomes in general (Watkins et al., 2015). There is thus a need to examine possible moderators to better understand the factors that may increase, or curb, the negative interpersonal impact of brooding rumination (Liu & Alloy, 2010).

## RSA and interpersonal functioning

Theorized as a psychophysiological marker of self-regulatory capacity (Balzarotti et al., 2017; Smith et al., 2020), RSA may moderate the association between brooding rumination and negative interpersonal behaviors. RSA, often measured using high-frequency heart rate variability, represents the fluctuations in time intervals between consecutive heartbeats associated with the respiration cycle (Berntson et al., 1997). Resting cardiac activity is slowed by the parasympathetic branch of the autonomic nervous system, which travels from the brain to the heart via the vagus nerve. This inhibition of cardiac activity is gated during inhalation and resumes during exhalation, periodically increasing and decreasing heart rate at the frequency of respiration. The magnitude of change in cardiac activity at the frequency of respiration corresponds to the magnitude of parasympathetic inhibitory signalling slowing the heart via the vagus nerve. The vagal parasympathetic output regulating RSA is modulated by brainstem nuclei that integrate input from cortical and limbic structures with input from sensory and visceral organs to coordinate cardiac activity with situational demands (Benarroch, 1993). Higher RSA is thought to reflect capacity for more flexible physiological responding to changing environmental demands (Berntson et al., 1997).

Two main conceptual models position RSA as a psychophysiological marker related to self-regulation in interpersonal relationships. Porges’ (2003) polyvagal theory is a phylogenetic model that posits that the autonomic nervous system evolved to support social behaviors in addition to regulating physiological arousal. This theory states facial muscles and sensory organs required for social engagement are co-regulated with the autonomic nervous system to coordinate somatic arousal, visual perception, audition, vocalization and facial gestures. Similarly, the neurovisceral integration model (Thayer & Lane, 2009) predicts the ability to effectively organize behaviors in response to situational demands is supported by greater concomitant prefrontal inhibition over limbic brain regions (e.g. amygdala) and related parasympathetic signalling toward the heart. Thus, these complementary conceptual models both predict the capacity for self-regulation in interpersonal contexts is rooted in a shared neurophysiological system that is indexed by resting levels of RSA.

Empirically, resting RSA has been associated with greater self-regulatory capacities (Balzarotti et al., 2017; Smith et al., 2020), like persistence on a difficult task (Segerstrom & Solberg Nes, 2007). Greater resting RSA has also been associated with different aspects of emotion regulation processes. Intrapersonally, RSA is related to less negative affect in response to stressors (Fabes & Eisenberg, 1997; Gouin et al., 2014), faster emotional recovery following stress (Diamond & Hicks, 2005; Souza et al., 2007) and the regulation of negative intrusive thoughts (Gillie et al., 2015). Interpersonally, greater RSA is related to better control of facial emotion expression (Tuck et al., 2016), emotion recognition (Quintana et al., 2012) and self-reported empathy (Lischke et al., 2018). Importantly, resting RSA is also associated with the maintenance of affiliative behaviors, even when experiencing negative affect (Gyurak & Ayduk, 2008). Within close relationships, higher RSA weakened the association between negative affect and negative social interactions (Diamond et al., 2011; Switzer et al., 2018), negative affective reactions to stress and marital quality over time (Ong et al., 2019) and reduced the impact of a marital partner’s brooding rumination on conflict within the couple (Caldwell et al., 2019). Greater RSA also reduced the impact of depressive symptoms on negative affect during social exchanges (Connell et al., 2011; 2015). These findings suggest that individuals with greater RSA may be better able to prevent brooding rumination from negatively affecting one’s interpersonal behaviors.

### Current studies

The current work evaluated the role of RSA as a moderator of the negative interpersonal consequences of brooding rumination. In three convenience samples, this study examined the interaction between brooding rumination and RSA on negative interpersonal behaviors and perception of received social support (Study 1), objectively rated chronic interpersonal stress (Study 2) and daily stressful interpersonal experiences (Study 3). The general hypothesis, across studies, is that higher levels of brooding rumination will be associated with more negative interpersonal behaviors and interpersonal stress, and that these associations will be stronger among individuals with lower RSA.

## Study 1

Nolen-Hoeksema et al. (2008) hypothesized brooding rumination is associated with negative interpersonal behaviors that erode social relationships. The negative interpersonal behaviors are hypothesized to represent maladaptive attempts to interpersonally regulate the negative *intra*personal experience of brooding. For example, brooding rumination prolongs negative affect, motivating individuals to seek excessive reassurance (Joiner et al., 1992). Empirically, brooding rumination has been associated with a range of negative interpersonal behaviors and processes, including excessive reassurance seeking, reduced praise, rejection sensitivity, submissive interpersonal style, and reactive aggression (Pearson et al., 2010, 2011; Baer and Sauer, 2011; White & Turner, 2014; Starr, 2015; Douglas et al., 2017; Stroud et al., 2018). While theorized as part of the same maladaptive interpersonal emotion regulation style (Nolen-Hoeksema et al., 2008; Pearson et al., 2011), prior work has examined different, yet conceptually-related, negative interpersonal behaviors independently. The current work replicates and extends previous findings by providing further evidence of an association between brooding rumination and a collection of negative interpersonal behaviors that have been empirically associated with brooding rumination and by examining the moderating role of RSA, a biomarker of self-regulatory capacity within interpersonal relationships.

Nolen-Hoeksema et al. (2008) also theorized brooding rumination may interfere with social support. Although the tendency to engage in brooding rumination is fairly stable over time (Bagby et al., 2004), its effect on perceived social support should be most evident when individuals are ruminating. The current analysis expands on previous findings by considering the relationship between brooding rumination and perceptions of received support on days when individuals report ruminating more often. This builds on previous work, which has solely considered rumination as a trait-like variable (Nolen-Hoeksema & Davis, 1999). Further, given ruminators’ tendency to perceive problems as overwhelming, generate less effective solutions to their problems and their low confidence in the efficacy of potential solutions (Nolen-Hoeksema et al., 2008), they may be particularly unlikely to seek instrumental support, compared to emotional support, on days when they are ruminating more than is usual for them. Accordingly, the following hypotheses were tested:

**H1:** Trait brooding rumination is positively associated with negative interpersonal behavior, and this association is increased among individuals with lower RSA.
**H2:** Daily increases in reported rumination are positively associated with perceptions of instrumental and emotional support; and greater trait brooding rumination, and lower RSA, reduces the association between daily rumination and perceived instrumental support.

## Method

### Participants

A sample of 153 female undergraduate students (age: mean = 21.76, median = 22.00, range = 18–29, SD = 1.94) in Montréal, Canada, gave informed consent and participated in exchange for course credit as part of a larger study on interpersonal exchanges among women. Given potential gender differences in the frequency (Johnson & Whisman, 2013) and negative interpersonal impact (McLaughlin & Nolen-Hoeksema, 2012) of rumination, we recruited a female sample. Exclusion criteria were taking medication affecting cardiac functioning (e.g. beta blockers) and smoking more than one cigarette per day. Approximately 45% of the participants were in a committed relationship. The distribution of self-reported ethnicities was 59.5% White, 10.5% Middle Eastern, 7.2% Asian, 5.2% South Asian, 4.6% Black, 2.6% Latino and 10.5% others.

### Procedures

All participants attended a laboratory session for RSA assessment. Participants were asked to refrain from strenuous exercise and the consumption of caffeine, alcohol and tobacco in the 2 hours prior to a laboratory session scheduled between 12 p.m. and 5 p.m. to attenuate exogenous and diurnal confounds. Upon arrival, three electrodes were fitted to participants in a Lead II configuration (right clavicle and bilaterally at bottom of ribcage) for the ECG recordings and they were seated in a comfortable chair. The research assistant then left the room and a computer screen prompted the participant to begin a 5-minute resting period where they were asked to sit upright, breathe normally and relax as much as possible without falling asleep while cardiac activity was recorded. Participants remained seated for the duration of the task to limit the influence of postural changes on RSA measurement. Following the session, participants completed a questionnaire assessing trait variables. Participants then filled out an electronic diary (using the same link to a secure Web site) to assess daily state rumination, instrumental support and emotional support every evening for 14 days. Participants completed an average of 12.45 entries (SD = 2.09).

### Measures

#### Brooding rumination

Brooding was assessed at baseline with the Ruminative Response Scale (Treynor et al., 2003). This instrument assessed the frequency individuals engage in moody pondering when they are feeling sad, down, or depressed (5 items; α = .84; *M* = 12.05; *SD* = 3.90; e.g. “think about a recent situation, wishing it had gone better”). Items were rated on a 4-point Likert-type scale ranging from 1 (*almost never*), to 4 (*almost always*).

#### Negative interpersonal behaviors

Problems in interpersonal functioning that have been associated with rumination in prior work were assessed at baseline using three measures. Excessive reassurance seeking was assessed with a subscale from the Depressive Interpersonal Relationships Inventory (Metalsky et al., 1991). This subscale includes four items (α = .93; e.g. “Do you frequently seek reassurance from the people you feel close to as to whether they really care about you?“) rated on a 7-point Likert-type scale from 1 (*not at all*) to 7 (*very much*). Salient interpersonal difficulties were assessed using the 32 items from the Inventory of Interpersonal Problems (Horowitz et al., 2000), all rated on a 5-point Likert-type scale from 0 (*not at all*) to 4 (*extremely*). This instrument includes seven subscales (domineering/controlling, vindictive/self-centered, cold/distant, socially avoidant/inhibited, non-assertive, over nurturant/over accommodating, exploitable/self-sacrificing and intrusive/needy), used to obtain a single global score (α = .92). Finally, anxious expectations of rejection in ambiguous social contexts were assessed using the Rejection Sensitivity Questionnaire (Downey & Feldman, 2013). This instrument asked participants to respond to eight scenarios (α = .74; e.g. “you go to a party and notice someone on the other side of the room and then you ask them to dance”) and to estimate their level of distress and the likelihood of rejection using two 7-point Likert-type scales. Within each scenario, the anticipated likelihood of rejection was reverse coded and then multiplied by that scenario’s distress score to create a summary rejection sensitivity score. Scores from these three questionnaires were converted to z-scores and averaged (M = 0; SD = 0.77, α = .66). Higher scores indicated more negative interpersonal behaviors.

#### State rumination

State rumination was assessed at baseline using the three-item mental capture subscale of the Perseverative Thinking Questionnaire (Ehring et al., 2011). Participants indicated the degree to which each statement corresponded to how they thought about past or future negative events on that day (e.g., “The same thoughts kept going through my mind again and again”) using a 5-point Likert-type scale ranging from 0 (*never*), to 4 (*always*). An average of 3.57 (*SD* = 2.34) within-person α = .98 and between-person α = 0.99 (Bonito et al., 2012) were reported.

#### Daily perceptions of instrumental and emotional support

Daily perceptions of received instrumental and emotional support were assessed nightly in reference to the following social entities: romantic partners, best friends, other friends, family and classmates or coworkers using a measure adapted from Otto et al. (2015) and Zautra et al. (2005). Participants responded to two questions, “Thinking about your social interactions today, which of the following individuals…” “did something concrete to help you deal with a problem?” and “listened to you and provided you with comfort?” Participants indicated if each social entity provided concrete help or comfort on that day. Participants could also select “No one” when appropriate. Each endorsed social entity was scored as 1, except for “No one,” which was scored as 0. The sum of endorsed social entities represented daily perceptions of received instrumental or emotional support (0–5 each day). An average of 1.18 (*SD* = 0.70), within-person α = .37 and between-person α = 0.96 for emotional support (Bonito et al., 2012); and an average of 0.67 (*SD* = 0.64), within-person α = .60 and between-person α = 0.94 for instrumental support (Bonito et al., 2012) were reported.

#### RSA

Cardiac data was collected using an ECG amplifier module within a Mindware Bionex 8-slot chassis (Mindware Technologies, Ltd, Gahanna, OH) at baseline. ECG signals were recorded continuously using a sampling rate of 1000 Hz. Mindware HRV Analysis software, Version 3.1, was used to analyze ECG recordings, detect improbable interbeat intervals using a validated automated algorithm (Berntson et al., 1997) and were then visually inspected and corrected when necessary. Fast Fourier Transformation was used to isolate the .15 to .40-Hz high frequency band of each 30-s epoch, which reflects the vagal-dependent parasympathetic influence on the heart, or RSA (Jarrin et al., 2012). Resting RSA was estimated as the mean value (natural log) of each 30-s epoch within the 5-minute resting period (*M* = 6.84, *SD* = 1.04).

## Results

### Between-person analysis of negative interpersonal behavior

**Figure 1.**
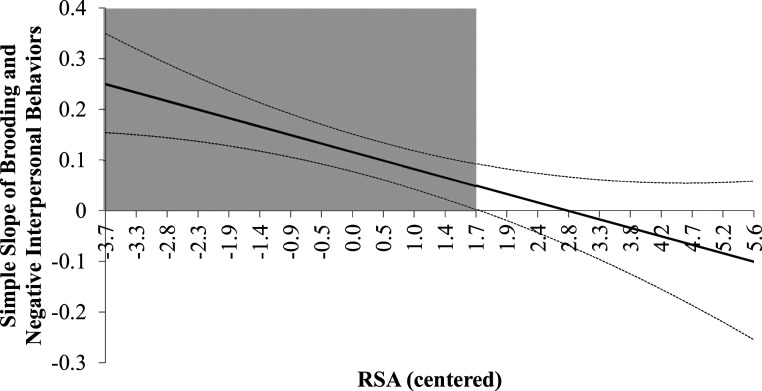
The Johnson-Neyman Procedure (1936) was used to estimate the point along the centered moderator (RSA) that the simple slope of the association between rumination and z-score transformed negative interpersonal behavior is significant (grey).

To test the hypotheses that brooding rumination would be associated with negative interpersonal behaviors and that this association would be attenuated among individuals with higher RSA, a hierarchical linear regression-based moderation model was estimated using the PROCESS macro (Preacher & Hayes, 2004) in SPSS version 20. To examine the specificity of RSA, we also examined whether brooding rumination interacted with resting heart rate (HR). The results are reported in Table 1. In the main effects model, brooding rumination was significantly and positively associated with negative interpersonal behaviors. The main effect of RSA was not statistically significant. In the moderation model, the interaction between brooding and RSA significantly predicted negative interpersonal behaviors and accounted for an additional 3.9% of the variance beyond that explained in the main effects model. The regions of significance are illustrated in Figure 1 and shows a stronger association between rumination and negative interpersonal behaviors when RSA was lower. A comparable simple slopes analysis is presented in Supplemental Appendix A. To examine the specificity of the moderation effect to RSA, a subsequent analysis found that a brooding by HR interaction term was not significant (*b* = .002, *p* = .12, 95% CI = −.0005 to .005).

**Table 1.** Moderation of the Association between Negative Interpersonal Behaviors and Brooding Rumination by RSA (Study 1).

			95% C.I.				95% C.I.	
Variables	b	*p*	Lower	Upper	b	*p*	Lower	Upper
Intercept	−1.032	.100	−2.265	.200	.227	.553	−.529	.984
HR	−.002	.737	−.012	.009	−.003	.566	−.013	.007
Brooding	.110	<.001	.083	.137	.112	<.001	.086	.139
RSA	−.023	.667	−.129	.083	−.021	.693	−.124	.083
Brooding x RSA					−.038	.004	−.063	−.013

*Note.* RSA = resting respiratory sinus arrhythmia; HR = resting heart rate.

### Within-person analysis of social support

The daily within-person associations between rumination and perceptions of received instrumental and emotional support were examined using multilevel modeling in order to account for the hierarchical structure (i.e. days nested within people) and serial dependency of daily diary data (Bolger & Laurenceau, 2013). The model was adjusted for serial dependency using a first-order auto-regressive covariance structure (Affleck et al., 1999). Further, a random effect was specified for the intercept and slope of the associations between perceived support and person-mean centered rumination. Cross-level interactions between person-mean centered rumination, trait brooding rumination and RSA were tested. Consistent with prior recommendations, associations were examined while controlling for the between-person centered (i.e., grand-mean centered; GMC) average of daily rumination (Howard, 2015). The between-person centered variable serves as a control for between-person differences in the amount of rumination that occurred over the study period. Theoretically, controlling for the average level of rumination helps clarify the effect of within-person, daily fluctuations in rumination (person-mean centered; PMC). Within-person differences in rumination were calculated by subtracting each individual’s own average daily rumination across the daily diary period from each day’s score to create a PMC rumination variable (i.e. daily deviations from that person’s own average). Thus, the interaction between an individual’s brooding personality style and their daily deviations from their own average level of rumination on a given day (PMC) represents a metric that will be predictive of the interpersonal consequences of individuals that are prone to maladaptive rumination (trait brooding), on days when they are engaged in more rumination than is normal for them (PMC rumination). The pseudo-R^2^ method was used to quantify the proportion of random slope variance explained when cross-level interactions were added to the models (Segerstrom & Solberg Nes, 2007). Statistical analyses were conducted using SAS PROC MIXED, version 9.4 (Cary, North Carolina, USA). The interaction between brooding rumination and HR was also tested to examine the specificity of the RSA effect.

In the main effects model, the association between PMC rumination and emotional support was significant, showing higher levels of PMC rumination were associated with more perceived emotional support (Table 2). The variance of the random slope reflecting the effects of PMC rumination on emotional support was not significant, suggesting little within-person variability in this association. Further, the results from the model including the interactions between PMC rumination, brooding and RSA, revealed no statistically significant interaction in the prediction of emotional support.

**Table 2.** Between- and Within-Person Effects of State Rumination on Daily Perceived Emotional Support (Study 1).

			95% C.I.				95% C.I.				95% C.I.	
	b	*p*	Lower	Upper	b	*p*	Lower	Upper	b	*p*	Lower	Upper
***Fixed effects***												
*Between-Person*												
Intercept	1.187	<.0001	1.076	1.298	1.187	<.0001	1.076	1.298	.871	.043	.027	1.715
State rumination (GMC)	.001	.969	−.047	.049	.000	.991	−.053	.053	.001	.979	−.054	.056
Brooding					.001	.936	−.031	.033	.003	.937	−.029	.036
RSA									−.036	.534	−.149	.077
HR									.004	.456	−.007	.016
Brooding x RSA									−.008	.567	−.036	.020
*Within-Person*												
State rumination (PMC)	.020	.039	.001	.039	.023	.025	.003	.042	.025	.014	.005	.045
*Cross-Level Interactions*												
State rumination (PMC) x brooding					−.003	.335	−.008	.003	−.003	.270	−.008	.002
State rumination (PMC) x RSA									−.002	.876	−.021	.018
State rumination (PMC) x brooding x RSA									−.002	.549	−.007	.004
			95% C.I.				95% C.I.				95% C.I.	
	Estimate	*p*	Lower	Upper	Estimate	*p*	Lower	Upper	Estimate	*p*	Lower	Upper
***Random effects***												
Intercept	.416	<.0001	.326	.551	.416	<.0001	.326	.551	.413	<.0001	.323	.548
Slope	.002	.142	.000	.041	.002	.129	.001	.033	.002	.161	.000	.064
Autoregressive structure	.118	<.0001	.062	.174	.119	<.0001	.063	.175	.119	<.0001	.063	.176

*Note.* GMC = grand-mean centered; RSA = resting respiratory sinus arrhythmia; HR = resting heart rate; PMC = person-mean centered.

In the main effects model the association between PMC rumination and perceived instrumental support was not statistically significant, suggesting a lack of within-person association (Table 3). The variance of the random slope reflecting the effect of PMC rumination on instrumental support revealed significant within-person variability in this association. Results from the models including the interactions between PMC rumination, brooding and RSA are also reported in Table 3. These results first revealed a significant two-way interaction between PMC rumination and trait brooding in the prediction of instrumental support, and accounted for an additional 9.84% slope variance relative to the main effects model. Consistent with the prediction that brooding rumination interferes with instrumental support, simple slopes analyses indicated higher than average within-person levels of PMC rumination were associated with more instrumental support for individuals reporting lower levels of trait brooding rumination (*b* = .035, *p* ≤ .01, 95% CI = .008 to .062), but not for individuals reporting higher levels of trait brooding rumination (b = −.008, *p* = .49, 95% CI = −.029 to .014).

**Table 3.** Between- and Within-Person Effects of State Rumination on Daily Perceived Instrumental Support (Study 1).

			95% C.I.				95% C.I.				95% C.I.	
	b	*p*	Lower	Upper	b	*p*	Lower	Upper	b	*p*	Lower	Upper
***Fixed effects***												
*Between-Person*												
Intercept	.667	<.0001	.566	.769	.666	<.0001	.566	.767	.679	.081	−.086	1.443
State rumination (GMC)	.033	.140	−.011	.076	.050	.043	.002	.097	.046	.068	−.003	.096
Brooding					−.026	.080	−.054	.003	−.023	.127	−.053	.007
RSA									−.039	.459	−.141	.064
HR									.000	.975	−.010	.010
Brooding x RSA									−.006	.619	−.032	.019
*Within-Person*												
State rumination (PMC)	.01	.258	−.007	.026	.014	.111	−.003	.031	.017	.044	.000	.033
*Cross-Level Interactions*												
State rumination (PMC) x brooding					−.005	.017	−.010	−.001	−.006	.010	−.010	−.001
State rumination (PMC) x RSA									.015	.066	−.001	.030
State rumination (PMC) x brooding x RSA									−.005	.030	−.009	.000
			95% C.I.				95% C.I.				95% C.I.	
	Estimate		Lower	Upper	Estimate		Lower	Upper	Estimate		Lower	Upper
***Random effects***												
Intercept	.355	<.0001	.279	.467	.348	<.0001	.273	.458	.350	<.0001	.275	.461
Slope	.002	.054	.001	.012	.002	.072	.001	.013	.001	.210	.000	.210
Autoregressive structure	.147	<.0001	.090	.204	.147	<.0001	.090	.204	.136	<.0001	.079	.193

*Note.* GMC = grand-mean centered; RSA = resting respiratory sinus arrhythmia; HR = resting heart rate; PMC = person-mean center

**Figure 2.**
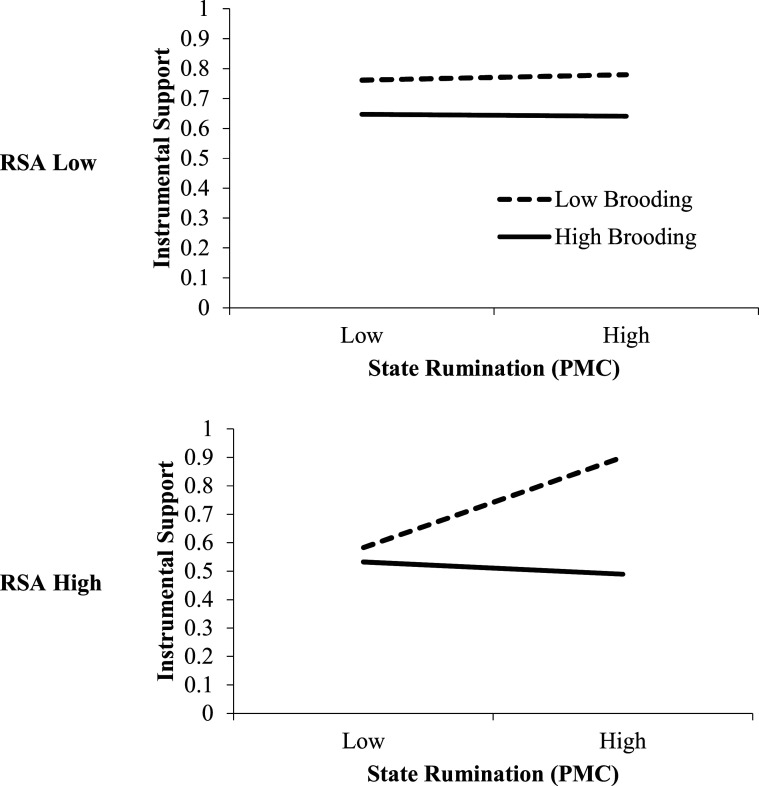
Interaction of brooding rumination, respiratory sinus arrhythmia (RSA) and person-mean centered state rumination on daily instrumental support (Study 1). Low and high represent +/− 1 SD of the mean.

Finally, the three-way interaction model between PMC rumination, trait brooding rumination and RSA was also statistically significantly associated with perceptions of received instrumental support. This interaction is graphically depicted in Figure 2. Simple slopes analyses indicated that at higher levels of RSA, higher levels of PMC rumination were associated with higher levels of instrumental support for participants reporting lower levels of trait brooding rumination (*b* = .074, *p* ≤ .0001, 95% CI = .036 to .111), but not for those reporting higher levels of trait brooding (*b* = −.010, *p* = .48, 95% CI = −.038 to .018). In contrast, at lower levels of RSA, no association was found between PMC rumination and instrumental support, regardless of the level of trait brooding rumination. The three-way moderation model accounted for an additional 45.37% slope variance compared to two-way moderation. This pattern indicates that perceptions of received instrumental support are more strongly associated with PMC rumination for individuals with a combination of lower trait brooding rumination and higher RSA. To examine the specificity of the moderation effect to RSA, a subsequent analysis found that an interaction term of brooding rumination with HR was not significant (*b* = −.0008, *p* = .844, 95% CI = −.0094 to .0077).

### Specific discussion: Study 1

Results from this first study showed, at the between-person level, individuals that endorsed more trait brooding rumination also reported more negative interpersonal behaviors and this effect was attenuated for individuals with higher levels of RSA. This finding is consistent with the hypotheses that the prolonged negative affective experience associated with brooding rumination may promote negative interpersonal behaviors (Nolen-Hoeksema et al., 2008) and that individuals with greater resting RSA may be better able to regulate their social behaviors within this context (Porges, 2003). Nonetheless, the full buffering effect of RSA may be limited to individuals that are exceptionally well-regulated, as the simple slope between rumination and negative interpersonal behaviors was primarily mitigated for individuals far above the mean of RSA. Further, at the within-person level, individuals that engaged in more than typical daily rumination (for them) reported greater perceptions of received emotional and instrumental support. However, trait brooding rumination and RSA selectively moderated the association between greater than typical daily rumination and perceptions of received instrumental support, but not emotional support. Higher levels of perceived instrumental support were associated with elevated daily rumination only for individuals who engaged in lower levels of trait brooding rumination and who displayed higher levels of RSA. This specific effect on perceptions of received instrumental support may reflect tendencies for ruminators to vent and portray problems as unsolvable, which could interfere with the receipt of instrumental support, but not emotional support. However, the current self-report data cannot distinguish whether ruminators were offered less support, or whether they did not perceive or construe specific social interactions as being supportive. The former, indicating an interpersonal mechanism, and the latter indicating an intrapersonal mechanism. Together, the findings suggest that individuals characterized by higher levels of brooding rumination and higher levels of RSA tend to engage in less negative interpersonal behaviors and to perceive less instrumental support when they engage in higher levels of daily rumination.

## Study 2

Results from Study 1 replicate and extend prior work indicating that brooding rumination is associated with a range of negative interpersonal behaviors (Nolen-Hoeksema et al., 2008). Furthermore, they suggest that the association between brooding rumination and negative interpersonal behaviors may be stronger among individuals with lower RSA. These brooding-related negative interpersonal behaviors may in turn elicit more stressful interpersonal experiences (Nolen-Hoeksema et al., 2008). The stress generation hypothesis posits individual vulnerabilities, like brooding rumination, may cause chronic interpersonal stress, in part, by way of their associations with negative interpersonal behaviors (Hammen, 2003). Empirical evidence suggests brooding rumination is associated with greater interpersonal stress, with both self-report (Lam et al., 2003; McLaughlin & Nolen-Hoeksema, 2012) and interviewer-rated measures (Stroud et al., 2015; 2018). Interviewer-rated measures offer a standardized assessment of chronic interpersonal stress across major interpersonal domains that are less tainted by the subjective experience of the respondent. Study 2 examined the association between interpersonal stress and brooding rumination by testing the following hypothesis:

**H3:** Greater trait brooding rumination will predict greater interviewer-rated chronic interpersonal stress across relationship domains, and the association will be stronger for individuals with lower RSA.

## Method

### Participants

Participants were part of a convenience sample of 42 adults meeting DSM-5 criteria for insomnia disorder (APA, 2013) who were recruited from the community via posters, newspaper ads and radio ads, as part of a larger clinical trial in Montréal, Canada. Exclusion criteria included the presence of a chronic unstable medical condition, sleep disorders other than insomnia (e.g. sleep apnea syndrome with apnea-hypopnea index greater than 5/h), severe mental illness (e.g. psychotic disorders, bipolar disorders, or substance use disorder), excessive alcohol use (>10 drinks/week) or illicit drug use (>1/month), chronic use of a hypnotic medication, cognitive impairment (<26 on the MOCA; Nasreddine et al., 2005), or employment involving nightshifts in the past year or during the study. On average, participants were 52.29 years old (median = 54.50, range = 23–82, SD = 15.96), 81% were female and 54.8% were married and living with their partner. About 83.3% of the sample self-reported their ethnicity as White, 7.2% Asian, 2.4% Black, and 4.8% identified as Other. 29.3% of the sample had a university degree or higher.

### Procedure

In the morning following an overnight visit, participants were fitted with electrodes in a Lead-II configuration for ECG recording. RSA was assessed using a Somnoscreen (Somnomedics GmbH, Randersacker, Germany) polysomnographic device during a 5-minute resting period (see Study 1). Participants also underwent an acclimatization period of several minutes prior to RSA assessment. Participants also completed a semi-structured interview assessing chronic stress exposure.

### Measures

#### Brooding rumination

Trait brooding rumination was assessed using the Ruminative Response Scale, as described in Study 1 (*M* = 10.00; *SD* = 2.38; α = .75).

#### Chronic interpersonal stress

Interpersonal stress was assessed using the chronic stress portion of the UCLA life stress interview (e.g. Adrian & Hammen, 1993; Hammen, 2003; Hammen, 2003). The interview questions assessed chronic stress over the previous 6 months across various life domains, including three interpersonal domains (social life, intimate relationships and family relationships). Consistent with previous work, each domain was coded by the interviewer using pre-defined anchor points that were described in behavioral terms (e.g. is support mutual in this relationship?) using a 5-point ordinal scale. The average of the three interpersonal domains was used in the current analysis (*M* = 1.68; *SD* = .61). Higher scores represent worse chronic interpersonal circumstances (e.g. more isolation, more conflict, less warmth and trust), which are deemed to be sources of stress for the respondents. The interviews were conducted by six trained interviewers and approximately 10% of the sample was re-rated by an independent coder to calculate inter-rater reliability (κ = .93).

#### RSA

Cardiac data were collected using an ECG amplifier via a Somnoscreen Stationary/Sleep Lab PSG and the Domino sleep diagnostic software suite (Somnomedics GmbH, Randersacker, Germany). ECG signals were recorded continuously using a sample rate of 512 Hz. Recording artifacts were manually edited and RSA was calculated using the procedure from Study 1 by two independent rating dyads (ICC = .97). Resting RSA was estimated as the mean value of each 60-s epoch within the 5-minute resting period (*M* = 5.41, *SD* = 1.25).

## Results

**Figure 3.**
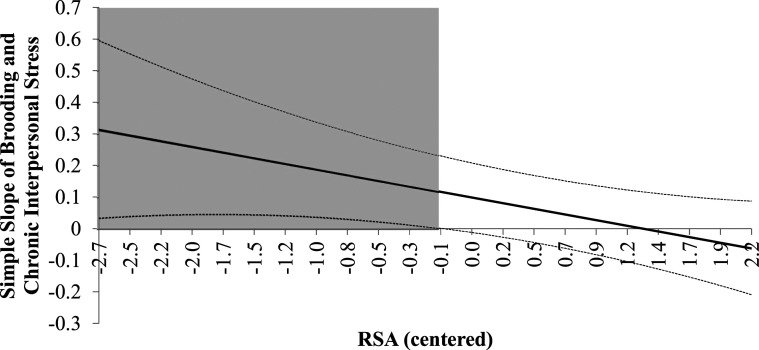
The Johnson-Neyman Procedure (1936) was used to estimate the point along the centered moderator (RSA) that the simple slope of the association between rumination and chronic interpersonal stress is significant (grey).

The hypothesis that greater brooding rumination is associated with greater chronic interpersonal stress and higher levels of RSA mitigate the association was examined using the same analytic strategy as Study 1. The moderation analyses were estimated while controlling for resting heart rate, age and sex (coded as 0-male, 1-female). In the main effects model, brooding rumination and RSA did not have significant main effects on interpersonal stress (Table 4). However, the results from the moderation model revealed a statistically significant interaction between brooding and RSA in the prediction of chronic interpersonal stress (*b* =-.057, *p* =.042, 95% CI = −.111 to −.002). The regions of significance are illustrated Figure 3 and shows a stronger association between rumination and chronic interpersonal stress when RSA was lower. A comparable simple slopes analysis is presented in Supplemental Appendix A. To examine the specificity of the moderation effect to RSA, a subsequent analysis found that a brooding rumination by HR interaction term was not significant (*b* =.0002, *p* = .12, 95% CI = −.0006 to .00,023).

**Table 4.** Moderation of the Association between Chronic Interpersonal Stress and Brooding Rumination by RSA (Study 2).

			95% C.I.				95% C.I.	
Variables	b	*p*	Lower	Upper	b	*p*	Lower	Upper
Intercept	3.565	.028	.197	6.452	3.089	.553	−.529	.984
Brooding	.069	.141	−.019	.162	.089	.069	−.007	.186
RSA	−.132	.263	−.334	.146	−.084	.489	−.327	.159
Brooding x RSA					−.057*	.042	−.111	−.002
HR	−.021	.235	−.057	.014	−.017	.373	−.055	.021
Age	−.001	.847	−.015	.015	.004	.634	−.012	.019
Female	−.178	.446	−.681	.305	−.203	.456	−.749	.343

*Note.* RSA = resting respiratory sinus arrhythmia; HR = resting heart rate.

### Specific discussion: Study 2

Findings from Study 2 indicate that brooding rumination is associated with interviewer-rated chronic interpersonal stress, but mostly among individuals that have resting RSA below the sample mean. The findings are consistent with prior empirical work reporting an association between brooding rumination and interpersonal stress in some, but not all studies (Lam et al., 2003; McLaughlin & Nolen-Hoeksema, 2012; Stroud et al., 2015; 2018). The present results suggest that greater RSA and perhaps the related capacity to self-regulate in stressful contexts, may mitigate the impact of brooding rumination on chronic interpersonal stress. Importantly, the measure of interpersonal stress used in the present study was interviewer-rated. This suggests the interaction between RSA and brooding rumination is associated with more objective differences in interpersonal stress, that are not solely related to subjective, perceptual differences in self-rated interpersonal stress that could be impacted by either brooding rumination or RSA.

## Study 3

Individuals who engaged in more brooding rumination and had lower RSA reported more negative interpersonal behaviours in Study 1 and were deemed by external raters to experience more stressful interpersonal experiences in Study 2. The goal of Study 3 was to extend these findings by examining whether RSA moderated the indirect effect linking rumination to depressive symptoms via increased interpersonal stress. According to the stress generation hypothesis, chronically stressful circumstances that develop from risk factors like brooding rumination increase risk for future depressive episodes (Hammen, 2003). Empirical work has demonstrated the generation of interpersonal stress is predictive of later depressive symptoms (Liu & Alloy, 2010). Importantly, interpersonal stress has been shown to mediate the association between brooding rumination and depression in young adults (Flynn et al., 2010). However the link between interpersonal stress and less pathological forms of repetitive thoughts, like reflective rumination (Treynor et al., 2003), have not been empirically examined and are important to highlight the unique association between brooding rumination and interpersonal stress. Study 3 tested the following hypothesis:

**H4:** The indirect association between brooding rumination and depressive symptoms, via greater interpersonal stress, is greater for individuals with lower levels of RSA.

## Methods

### Participants

Participants were drawn from a larger project on individuals undergoing chronic caregiving stress because they are at the heightened risk for depressive symptoms (Lovell et al., 2012). Mothers of adolescents with (*n* = 125) and without (*n* = 97) developmental disorders (total *n* = 222) gave their informed consent to participate in a study on caregiving stress and health in Montréal, Canada. Exclusion criteria included chronic medical conditions, regular use of anti-inflammatory medication, major mental illness (e.g. schizophrenia, bipolar disorder, or substance misuse), or being pregnant or nursing at the time of the study. Mothers were recruited through advertisements via school boards, social service centers, community organizations, as well as general advertisements in local newspapers. On average, mothers were 46.83 (median = 47.00, range = 34–65, SD = 6.03) years old and had an adolescent that was 15.89 (SD = 2.5) years old. About 77.5% of the participants self-identified as White, 10.4% as Black, 9.1% as Asian, 0.9% as First Nations, 6.8% as Latin American, and 1.8% as Other. 30.6% had a university degree and 52.5% had a household family income below CAN$ 60,000. Approximately 76.2% were married or in a common-law relationship.

### Procedure

Participants first completed an online questionnaire assessing rumination and depressive symptoms. Next, all participants completed an online daily diary assessing interpersonal stress at the end of 7 consecutive days. During an in-person morning visit to the participants’ homes or at the university laboratory, participants were fitted with a chest belt hardwired with a digital inter-beat interval recorder (Polar RS800CX; Finland: Kempele). They completed a 5-minute resting period during which they were asked to sit upright, relax and breathe normally without speaking to assess resting RSA. Participants also underwent an acclimatization period of several minutes prior to RSA assessment.

### Measures

#### Rumination

Rumination was assessed with the Ruminative Response Scale (Treynor et al., 2003). This questionnaire incorporates two 5-item subscales assessing brooding rumination (described in Study 1; α = .77; *M* = 10.98; *SD* = 3.28) and reflection (e.g. “go someplace alone to think about your feelings”; α = .78; *M* = 10.92; *SD* = 3.20).

#### Interpersonal stress

On seven consecutive evenings, participants responded to the question (adapted from the Daily Inventory of Stressful Events; Almeida et al., 2002), “how much stress or tension did you experience in your interactions with the following people?” followed by a 4-point Likert-type scale from 0 (*not at all*) to 3 (*extremely*) for each interpersonal domain: family, friends, partner and coworkers. The mean score across 7 days was taken to reflect interpersonal stress in each relationship domain. Participants completed an average of 5.06 (*SD* = 1.96) days. Average interpersonal stress reported was 1.51 (*SD* = .42) and scale score reliability was satisfactory (α = .88).

#### Depressive symptoms

Depressive symptoms were measured using the Center for Epidemiological Studies Depression Scale (CES-D; Radloff, 1977). This 20-item scale assesses depressive symptoms over the previous week. Responses were provided on a 4-point Likert-type scale from 1 (*rarely or none of the time (less than 1-day)*) to 4 (*most or all of the time (5-7 days)*). The CES-D includes four subscales assessing depressed affect (7 items, e.g., “I felt depressed”), low positive affect (4 items, reverse coded, e.g., “I was happy”), somatic complaints (7 items, e.g., “I did not feel like eating; my appetite was poor”) and interpersonal problems (2 items, e.g., “I felt that people dislike me”). In the present study, we rely on a total depression score encompassing the first three subscales (α = .90; *M* = 15.49; *SD* = 10.22). The interpersonal problems subscale was not included in the CES-D total score to reduce conceptual overlap between the estimates of interpersonal stress and depression.

#### RSA

Cardiac data was collected using a mobile chest belt and digital inter-beat interval recorder (Polar RS800CX; Finland, Kempele). Interbeat intervals were recorded continuously using a sampling rate of 1000 Hz. Recording artifacts were corrected in CardioEdit software (2007) using integer arithmetic (i.e. adding or dividing). Porges et al.’s (1980) moving polynomial approach was used to extract RSA using CardioBatch software (2007). Resting RSA was estimated as the mean value (natural log) of all 30-s epochs within the 5-minute resting period (*M* = 5.63, *SD* = 1.34).

### Analyses

Analyses were conducted using the MPlus 8.3 statistical package (Muthén & Muthén, 2019). Confirmatory factor analysis (CFA) and structural equation modeling (SEM) were used to examine whether higher levels of interpersonal stress mediated the relation between brooding rumination and depressive symptoms and whether RSA moderated this indirect pathway. The specific details of the CFA model building procedure can be found in Supplemental Appendix B.

## Results

**Figure 4.**
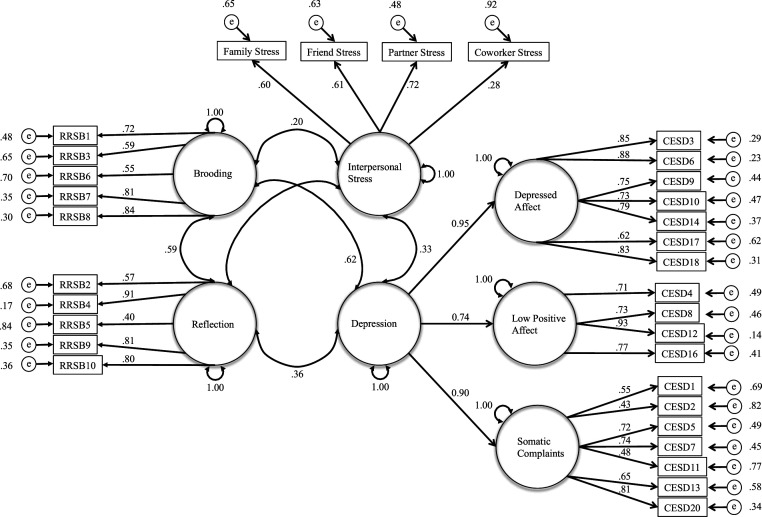
Measurement model with standardized uniquenesses, disturbances, factor loadings, factor variance and higher order factor correlations. Note. Circles represent latent variables, rectangles represent latent indicators, double headed arrows represent correlations, single headed arrows represent factor loadings and small circles marked by an *e* and linked with an arrow represent items uniquenesses. All factor loadings significant at *p* ≤ .01. All correlations depicted in the model were significant at *p* ≤ .01. Non-significant correlations are not depicted.

The results from the measurement model, depicted in Figure 4, revealed the latent measurement model achieved an excellent level of fit to the data (χ^2^ = 674.361, *p* < .01; RMSEA = .047; CFI = .954; and TLI = .950) and resulted in well-defined factors.

The first predictive model achieved an excellent level of fit to the data (χ^2^ = 20.415, *p* = .20; RMSEA = .035; CFI = .982; and TLI = .972). The results from this model revealed a significant positive association between brooding rumination and interpersonal stress and a negative association between reflective rumination and interpersonal stress (Table 5, left). Significant direct associations were observed between depression and brooding rumination, interpersonal stress, as well as caregiving status. The indirect effect of brooding rumination on depression via interpersonal stress was also significant (estimate = .074, *SE* = .036, *p* = .041, bootstrapped 95% C.I. = .019 to .197). This indirect pathway accounted for approximately 10.4% of the total effect of brooding on depression, via interpersonal stress. The indirect effect of reflective rumination on depression via interpersonal stress was not significant (estimate = −.051, *SE* = .030, *p* =.09, bootstrapped 95% C.I. = −.158 to −.008).

**Table 5.** Mediation and Moderated Mediation Models of the Association between Depression and Brooding Rumination, via Interpersonal Stress, Moderated by RSA (Study 3).

	Mediation							Moderated mediation				
						95% bootstrapped C.I. of unstandardized estimate					95% bootstrapped C.I. of unstandardized estimate	
Variables	b	*p*	S.E.	β	*p*	Lower	Upper	b	*p*	S.E.	Lower	Upper
*Outcome: Interpersonal Stress*												
Caregiving status	−.022	.520	.035	−.055	.510	−.099	.042	−.031	.397	.037	−.113	.034
Brooding	.158	<.0001	.045	.357	<.0001	.072	.263	.163	.001	.048	.075	.287
Reflection	−.109	.017	.046	−.247	.011	−.220	−.027	2.676	.258	3.063	−3.010	10.819
RSA								−.03	.258	.027	−.098	.019
RSA*Brooding								−.06	.021	.026	−.127	−.01
HR								−2.532	.366	2.799	−9.962	2.596
*Outcome: Depression*												
Caregiving status	.125	.003	.042	.139	.003	.039	.207	.125	.003	.042	.042	.213
Interpersonal stress	.470	.017	.196	.211	.005	.122	1.101	.458	.025	.204	.107	1.32
Brooding	.636	<.0001	.070	.647	<.0001	.498	.783	.634	<.0001	.07	.476	.775
Reflection	.024	.704	.063	.024	.705	−.113	.149	.026	.685	.063	−.105	.154
*Correlation*												
Brooding with reflection	.537	<.0001	.063	.651	<.0001	.424	.664	.537	<.0001	.063	.422	.660

*Note.* Brooding, reflection and depression are factor scores generated from the measurement model; Interpersonal stress is a latent variable; Model 1 represents the path coefficients for the indirect effects model; Model 2 represents the path coefficients for the moderated indirect effect model; Unstandardized coefficients (b), standard errors (S.E.) and standardized coefficients (β) are presented; 95% C.I. using 5000 bootstrapped samples; RSA = Respiratory sinus arrhythmia.

The results from the final moderated mediation model are reported in Table 5 (right). These results show the interaction between RSA and brooding rumination was a significant predictor of interpersonal stress, supporting the moderating role of RSA in the relation between brooding rumination and interpersonal stress. Simple slope analyses suggest the positive effect of brooding rumination was not significant at high levels of RSA (*b* = .083, *SE* = .049, *p* =.087, bootstrapped 95% CI = −.008 to .209), but was significant at low levels of RSA (estimate = .242, SE = .068, *p* ≤ .001, bootstrapped 95% CI = .116 to .418). When these simple slopes are considered within the context of the indirect relation between brooding and depression, mediated by interpersonal stress, the results further show the indirect effect is not statistically significant at higher levels of RSA (estimate = .038, SE = .028, *p* = .171, bootstrapped 95% CI = −.001 to .164), but was significant at lower levels of RSA (estimate = .111, *SE* = .050, *p* = .027, bootstrapped 95% CI = .029 to .291). To examine the specificity of the moderation effect to RSA, a subsequent analysis found that a brooding by HR interaction term was not significant (*b* = .029, *p* = .404, 95% CI = −.039 to .097).

### Specific discussion: Study 3

The results from study 3 revealed a positive indirect effect of brooding rumination on depression via daily interpersonal stress. This indirect effect was moderated by RSA, such that the effect of brooding rumination via interpersonal stress was non-significant for individuals with higher levels of RSA. These results thus supported the stress generation hypothesis, suggesting brooding rumination is associated with depressive symptoms via associations with interpersonal stress (Hammen, 2003). These results replicate prior work showing interpersonal stress mediates the association between rumination and depression (Flynn et al., 2010) in an adult community sample. Furthermore, the indirect path from brooding rumination to depression via interpersonal stress was moderated by RSA, showing higher levels of RSA mitigated the negative interpersonal consequences of brooding rumination. The results thus support the conceptualization of RSA a marker associated with self-regulatory capacity in interpersonal relationships (Porges, 2003) and highlight the potential importance of self-regulatory capacity in attenuating the stressful interpersonal experiences by which brooding rumination and depressive symptoms are associated.

## General discussion

The current research examined the moderating role of RSA, a marker of self-regulatory capacity, in the association between brooding rumination and negative interpersonal behaviors, social support and interpersonal stress in three convenience samples. Higher levels of RSA were found to reduce the strength of the associations between brooding rumination and negative interpersonal behaviors and daily instrumental support (Study 1), interviewer-ratings of chronic interpersonal stress (Study 2) and daily average interpersonal stress (Study 3). The findings support the hypothesis that among individuals with greater RSA, the association between brooding rumination, a maladaptive intrapersonal emotion regulation strategy, and associated negative interpersonal outcomes was attenuated, compared to their counterparts with lower RSA.

Increasing evidence shows intrapersonal emotion regulation impacts interpersonal processes (Zaki & Craig Williams, 2013). Previous work has demonstrated maladaptive and adaptive coping strategies tend to have opposing effects on interpersonal outcomes (Butler et al., 2003; Richards et al., 2003). Conceptualizing brooding rumination as a maladaptive emotion regulation strategy, our findings converge in demonstrating that rumination is associated with negative interpersonal behaviors (Study 1), reduced instrumental support (Study 1) and increased interpersonal stress (Studies 2 and 3). These findings extend prior work by indicating that ruminating in response to negative mood appears to negatively impact interpersonal emotion regulation by reducing instrumental support and by generating chronic interpersonal stress.

Brooding rumination has been associated with a host of maladaptive interpersonal behaviors (Nolen-Hoeksema et al., 2008) and impoverished social support resources (King & DeLongis, 2014; Nolen-Hoeksema & Davis, 1999). Previous work suggests ruminators perceive less emotional support (Nolen-Hoeksema & Davis, 1999) and elicit more withdrawal behaviors from their partners when they ruminate (King & DeLongis, 2014). The current work considers social support across relationships and differentiates emotional and instrumental support. In doing so, it adds nuance to previous findings by demonstrating that while daily fluctuations in rumination are associated with both instrumental and emotional support, instrumental support is selectively reduced among individuals with greater trait brooding rumination (Study 1). Brooding rumination may interfere with instrumental support because it is associated with perceiving problems as overwhelming, excessively venting about negative emotion, generating less effective solutions, reducing confidence in the efficacy of potential solutions and reducing instrumental behaviors (Nolen-Hoeksema, 2008). Thus, convergent with other work, brooding rumination is associated reduced emotional and instrumental support and increasing interpersonal stress in the current studies (Study 1; McLaughlin & Nolen-Hoeksema, 2012; Stroud et al., 2018).

Supporting the hypothesis that RSA is related to greater self-regulatory capacity within interpersonal contexts (Porges, 2003; Thayer & Lane, 2009), greater RSA appeared to attenuate the negative interpersonal consequences associated with brooding rumination, whether it was conceptualized as negative interpersonal behaviors (Study 1), interviewer-rated chronic stress (Study 2), or subjectively reported daily stress (Study 3). Greater RSA may assist individuals resist urges to perform negative interpersonal behaviors that are associated with negative affect and depressed mood (e.g. Diamond et al., 2011; Connell et al., 2011; 2015; Switzer, et al., 2018). The current research also suggests some of the inconsistent findings linking rumination to interpersonal stress generation (Hamilton et al., 2013, 2017; Shapero et al., 2013) may be partially explained by individual differences in RSA. Notably, this effect was specific to RSA as none of the brooding rumination by HR interactions were associated with interpersonal outcomes. However, the moderation effect of RSA was observed mostly for the participants exhibiting the highest RSA levels, likely those having the highest level of self-regulation capacities.

Finally, Study 3 (Affleck et al., 1999) supports the stress generation hypothesis of depression, which predicts that individual vulnerabilities, like brooding rumination, are associated with depressive symptoms via interpersonal stress (Hammen, 2003). The findings converge with prior work showing interpersonal stress and negative interpersonal behaviors mediate the association between brooding rumination and depressive symptoms (Flynn et al., 2010; Stroud et al., 2018). Furthermore, these stressful interpersonal experiences were moderated by RSA, suggesting individuals with greater RSA are better able to limit the effect of this maladaptive intrapersonal emotion regulation strategy on interpersonal behaviors. These findings highlight the potential importance of self-regulatory capacity, indexed by RSA, in preventing the spill-over of brooding-related negative affect into interpersonal exchanges, which may help prevent a cascade of inter-related interpersonal stress and depressive symptoms.

### Implications

These findings suggest that individual differences in parasympathetic functioning may be related to interpersonal emotion regulation, notably by attenuating the associations of greater brooding rumination with more negative interpersonal behaviors and greater interpersonal stress. This work represents theoretical and empirical integration across cognitive and psychophysiological areas of research that have developed independently, and converge within an interpersonal emotion regulation framework. Further, the interaction between rumination and RSA are reliable across diverse measures of interpersonal functioning and replicate in different convenience sample populations (healthy, at-risk and clinical), increasing our confidence in the presence of a rumination by RSA interaction effect. The convergence of these findings suggest that the consequences of intrapersonal emotion regulation may be better characterized within their interpersonal context.

### Limitations and future directions

The major limitation of the current set of studies is that each analysis was cross-sectional, precluding conclusions regarding the directionality of the associations. The stress generation hypothesis suggests greater rumination-related interpersonal stress generation will be associated with increased risk for depression over time (Hammen, 2003) and thus longitudinal models are needed to assess individual trajectories in interpersonal stress and depressive symptoms. Nonetheless, when supplementary analyses examining reverse causality and controls for perceived stress were evaluated (see Supplemental Appendix C), the models did not support these alternative interpretations of the cross-sectional data. Further, as expected, the main effect between within-person rumination and emotional support remains significant in the reverse causal model and is also not moderated by RSA.

The use of mostly white, female, undergraduate and clinical samples (i.e. insomnia disorder) may limit generalizability until replication in larger and more representative samples are conducted. Given potential gender differences in the frequency (Johnson & Whisman, 2013) and negative interpersonal impact (McLaughlin & Nolen-Hoeksema, 2012) of rumination, we note that female samples used in Studies 1 and 3 are an important limit to interpretation. We also note that gender and sexual identities were not addressed in the current set of analyses.

Additionally, while the interaction effect was conceptually replicated in three relatively small convenience samples, the relatively small sample size used in all of the analyses makes it particularly important to replicate the results using consistent measures of negative interpersonal behaviors and social support in larger more representative samples. Relatedly, the combination of questionnaires used to assess negative interpersonal behavior had an acceptable, but relatively low Cronbach’s alpha, probably indicating that the choice of measures to assess rumination-related interpersonal behavior was not optimal. Future work should clarify the theoretical and empirical description of rumination-related negative interpersonal behaviors. Future work should also focus on characterizing the specific self-regulatory processes related to higher RSA that buffer the association between brooding rumination and negative interpersonal behaviors. Finally, as respiration was not measured in the current samples, we cannot estimate the impact of respiration on the variance in RSA (Grossman & Taylor, 2007). Nonetheless, these concerns may be partially assuaged by meta-analytic findings that indicate the association between RSA and social functioning remains significant after adjusting for respiration (Graziano et al., 2013).

## Conclusion

The collective findings from this set of studies are consistent with an interpersonal perspective on emotion regulation and supports the hypothesis that brooding rumination is associated with negative interpersonal behaviours, less instrumental social support and more stressful interpersonal environments. Further the negative interpersonal consequences of brooding rumination are attenuated among individuals with greater RSA, a biomarker associated with better self-regulation in interpersonal relationships. These findings highlight the negative interpersonal consequences of brooding rumination, particularly among individuals with lower RSA.

## Supplemental Material

Supplemental Material - Respiratory sinus arrhythmia moderates the interpersonal consequences of brooding rumination Click here for additional data file. Supplemental Material for Respiratory sinus arrhythmia moderates the interpersonal consequences of brooding rumination by Warren Caldwell, Sasha MacNeil, Carsten Wrosch, Jennifer McGrath, Thanh Dang-Vu, Alexandre Morin and Jean-Philippe Gouin in Journal of Social and Personal Relationships.
