# Supplementary material for: Respiratory sinus arrhythmia moderates the interpersonal consequences of brooding rumination
Source: J Soc Pers Relat. 2022 Aug 19;40(2):624–53. doi: 10.1177/02654075221122059 (PMC9941653; doi:10.1177/02654075221122059)
Supplement: Supplemental Material - Respiratory sinus arrhythmia moderates the interpersonal consequences of brooding rumination [file sj-pdf-1-spr-10.1177_02654075221122059.pdf]

Appendix A: Supplementary Analyses and Notes

Simple Slopes Analyses

Study 1

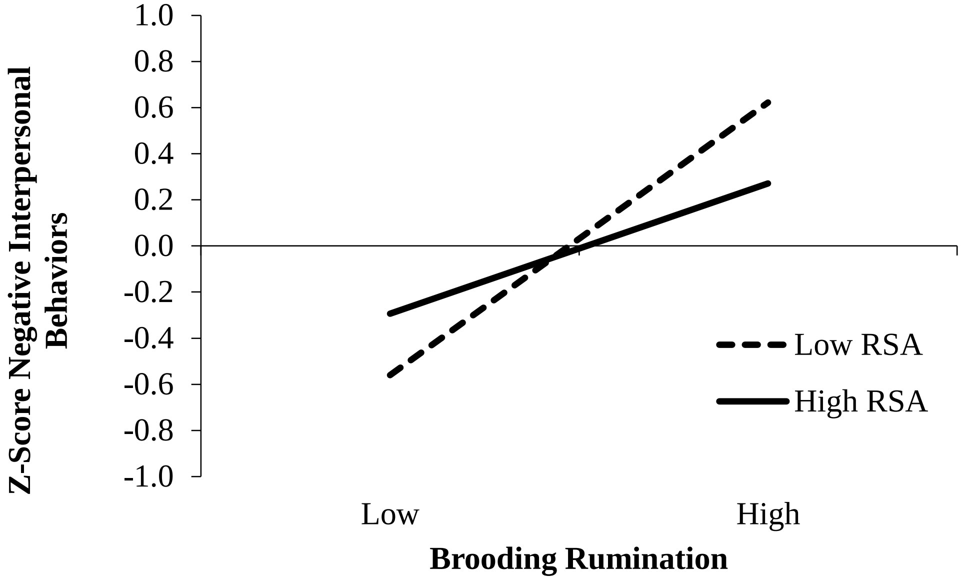

Interaction between brooding rumination and respiratory sinus arrhythmia (RSA) on z-score transformed negative interpersonal behavior (Study 1). Low and high represent +/- 1 SD of the mean. The effect of brooding on negative interpersonal behaviors was stronger when RSA was lower (b = .152, p < .0001, 95% CI = .113 to .190) compared to when RSA was higher (b = .070, p < .0001, 95% CI = .036 to .109)

Study 2

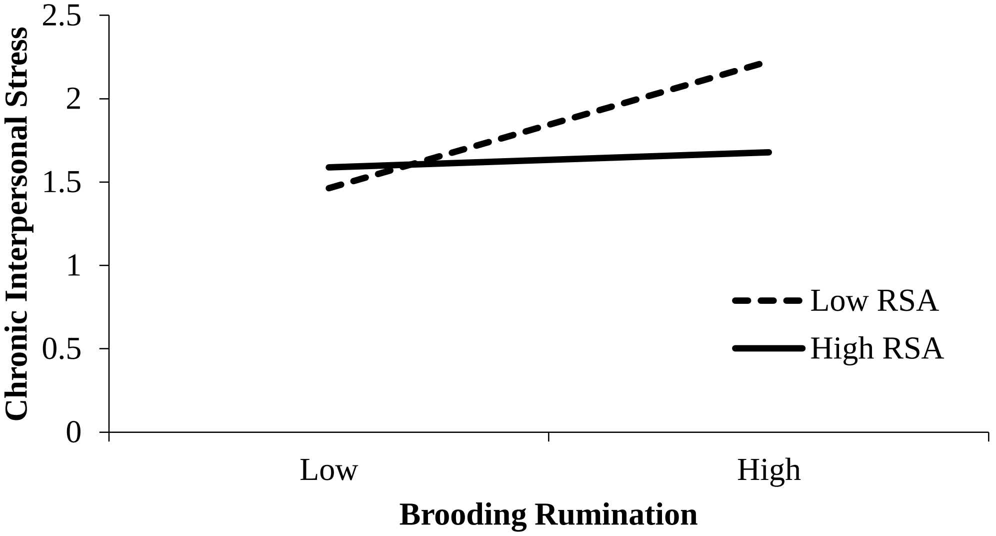

Interaction between brooding rumination and respiratory sinus arrhythmia (RSA) on chronic interpersonal stress (Study 2). Low and high represent +/- 1 SD of the mean. The effect of brooding rumination on chronic interpersonal stress was significant when RSA was low (b =

.160, *p* = .022, 95% CI = .032 to .288), but not when RSA was high (*b* = .019, *p* =.829, 95% CI = -.088 to .125).

Appendix B: Study 3 SEM Model Building Procedure

Consistent with previous work on the factor structure of the CES-D, a higher order CFA model was used to estimate a global (i.e. higher-order) depression factor defined by three (negative affect, low positive affect, somatic complaints) first-order factors (Morin et al., 2011). In order to account for the ordered categorical nature of the four-point Likert scales utilized for the assessment of brooding, reflection, and depression, these ratings were treated as ordinal variables. The resulting measurement model (CFA) was thus estimated using a robust (mean and variance adjusted) weighted least square estimator (WLSMV in Mplus), which is generally recommended for ordinal response scales incorporating fewer than 5 response categories (for a review, see Finney & DiStefano, 2013). Due to its different rating method, interpersonal stress was still estimated as a latent variable in the predictive models, maintaining a complete control for unreliability. Model fit was determined by examining the Confirmatory Fit Index (CFI), the Tucker Lewis Index (TLI), and the root mean square error of approximation (RMSEA) in order to compensate for the known oversensitivity of the chi-square test of exact fit to sample size and minor model misspecifications (Hu & Bentler, 1999; Marsh, Hau, & Grayson, 2005).

The role of interpersonal stress as a mediator of the association of depression on brooding and self-reflection was assessed via the estimation of indirect effects, estimated as the product of the two coefficients forming the mediation chain (MacKinnon, Lockwood, & Williams, 2004) and implemented in Mplus via the MODEL INDIRECT function (Muthén & Muthén, 2019). This model was estimated via the Robust Maximum Estimator, which provide standard error and tests of model fit that are robust to non-normality (Satorra & Bentler, 1994). The statistical significance of these indirect effects was calculated using bias-corrected bootstrapped 95% confidence intervals (CIs based on 5000 bootstrap samples; Cheung & Lau, 2008).

Given that latent interaction effects cannot yet be estimated using WLSMV estimation (Marsh, Hau, Wen, Nagengast, & Morin, 2013), factor scores, estimated in standardized units with a mean of 0 and a standard deviation of 1, were extracted from this measurment model in order to represent participants' scores of brooding, self-reflection, and depression. Although factor scores are unable to completely control for unreliability the way latent variables do, they still afford a partial control for measurement error (Skrondal & Laake, 2001), in addition to preserving the underlying nature of the measurement model, which was important to account for the higher order structure of the CES-D (e.g. Morin et al., 2016). Due to its different rating method encompassing ratings previously aggregated over seven days, interpersonal stress was still estimated as a latent variable in the predictive model (i.e., no factor score was necessary), maintaining a complete control for unreliability.

The moderated-mediation model was similar to the previous one and included RSA observed scores as an additional predictor. The interaction term was calculated as the product of the predictor (brooding rumination) and the moderator (RSA) (Marsh, Hau, Wen, Nagengast, & Morin, 2013). Simple slope analyses were implemented in Mplus via the model CONSTRAINT function (Hayes & Preacher, 2013).

Both predictive models (mediation and moderated-mediation) were estimated via the Robust Maximum Estimator (MLR), which provide standard error and tests of model fit that are robust to non-normality. For all models, acceptable model fit was determined by CFI and TLI

values exceeding .90 or RMSEA values below .08, whereas excellent model fit was reflected by values exceeding .95 or RMSEA values below .06 (Hu & Bentler, 1999; Marsh, Hau, & Grayson, 2005; Yu, 2002).

References

Cheung, G. W., & Lau, R. S. (2008). Testing mediation and suppression effects of latent variables: Bootstrapping with structural equation models. *Organizational Research Methods*, *11*, 296–325.

Finney, S. J., & DiStefano, C. (2013). *Non-normal and categorical data in structural equation modeling*. (J. R. Handcock & R. O. Mueller, Eds.) (2nd ed.). Greenwich, CO: IAP.

Hu, L., & Bentler, P. M. (1999). Cutoff criteria for fit indexes in covariance structure analysis: Conventional criteria versus new alternatives. *Structural Equation Modeling*, *6*, 1–55.

MacKinnon, D. P., Lockwood, C., & Williams, J. (2004). Confidence limits for the indirect effect: Distribution of the product and resampling. *Multivariate Behavioral Research*, *39*, 99–128.

Marsh, H. W., Hau, K.-T., & Grayson, D. (2005). Goodness of fit evaluation in structural equation modeling. In A. Maydeu-Olivares & J. Mcardle (Eds.), *Contemporary psychometrics: A Festschrift for Roderick P. McDonald*. Mahwah, NJ: Erlbaum.

Marsh, H. W., Hau, K.-T., Wen, Z., Nagengast, B., & Morin, A. J. S. (2013). Moderation. In T. D. Little (Ed.), *Oxford Handbook of Quantitative Methods* (2nd ed., pp. 361–386). New York, NY: Oxford University Press.

Morin, A. J.S., Moullec, G., Maïano, C., Layet, L., Just, J. L., & Ninot, G. (2011). Psychometric properties of the Center for Epidemiologic Studies Depression Scale (CES-D) in French clinical and nonclinical adults. *Revue d'Epidemiologie et de Sante Publique*, *59*(5), 327–340. https://doi.org/10.1016/j.respe.2011.03.061

Morin, A.J.S., Boudrias, J.S., Marsh, H.W., Madore, I., & Desrumaux, P. (2016). Further Reflections on Disentangling Shape and Level Effects in Person-Centered Analyses: An Illustration Exploring the Dimensionality of Psychological Health. *Structural Equation Modeling*, *23*(3), 438–454. https://doi.org/10.1080/10705511.2015.1116077

Satorra, A., & Bentler, P. (1994). Corrections to test statistics and standard errors in covariance structure analysis. In *Latent variables analysis: Applicaitons for developmental research* (pp. 399–419).

Skrondal, A., & Laake, P. (2001). Regression among factor scores. *Psychometrika*, *66*(4), 563–575. https://doi.org/10.1007/BF02296196

Appendix C: Reverse causality and additional covariate models to support the validity of the cross-sectional main findings

Study 1

To examine reverse causality, we substituted the predictor and outcome variables.

Between-person analysis:
Predicting brooding rumination

Effect	Estimate	p-value	Lower	Upper
Intercept	11.29	<.0001	7.37	15.22
RSA	.13	.62	-.41	.69
Negative Interpersonal Behaviors	2.69	.00	2.01	3.37
RSA*Negative Interpersonal Behaviors	-.60	.13	-1.40	.19
HR	.01	.71	-.04	.06

Within-person analysis:
Predicting daily total rumination

Effect	Estimate	p-value	Lower	Upper
Intercept	6.43	<.0001	4.05	8.81
Emotional Support (GMC)	-.04	.88	-.49	.43
Brooding	.28	<.0001	.19	.36
RSA	-.12	.47	-.45	.21
HR	-.04	.02	-.07	-.01
Brooding*RSA	-.08	.06	-.16	.01
Emotional Support (PMC)	.15	.03	.01	.28
Brooding*Emotional Support (PMC)	-.01	.61	-.04	.02
RSA*Emotional Support (PMC)	-.005	.94	-.13	.12
Brooding*RSA*Emotional Support (PMC)	-.014	.38	-.05	.02

Predicting daily total rumination

Effect	Estimate	p-value	Lower	Upper
Intercept	6.45	<.0001	4.07	8.82
Instrumental Support (GMC)	.41	.10	-.09	.92
Brooding	.28	<.0001	.20	.36
RSA	-.10	.52	-.43	.22
HR	-.04	.02	-.07	-.01
Brooding*RSA	-.07	.07	-.15	.01
Instrumental Support (PMC)	.07	.34	-.08	.23
Brooding* Instrumental Support (PMC)	-.04	.04	-.08	-.01
RSA* Instrumental Support (PMC)	.08	.39	-.10	.25

Brooding*RSA* Instrumental Support (PMC)	-.03	.13	-.07	.01

To start to address the possibility that stress could be a potential third variable, we ran additional analyses examining if the findings are robust when daily subjective stress was included as a covariate in the model to controlling for the associations among perceived stress, support and rumination.

Predicting Emotional Support

Effect	Estimate	p-value	Lower	Upper
Intercept	.90	.04	.06	1.74
State Rumination (GMC)	-.01	.68	-.07	.04
Brooding	-.001	.94	-.03	.03
RSA	-.03	.57	-.14	.08
HR	.003	.50	-.007	.02
Brooding*RSA	-.008	.55	-.04	.02
State Rumination (PMC)	.02	.12	-.004	.04
Brooding*State Rumination (PMC)	-.003	.24	-.01	.002
RSA*State Rumination (PMC)	-.0007	.94	-.02	.02
Brooding*RSA*State Rumination (PMC)	-.002	.48	-.007	.003
Subjective stress (GMC)	.08	.13	-.02	.18
Subjective stress (PMC)	.03	.01	.006	.05

Predicting Instrumental Support

Effect	Estimate	p-value	Lower	Upper
Intercept	.72	.06	-.03	1.47
State Rumination (GMC)	.03	.32	-.03	.08
Brooding	-.03	.05	-.06	-.0002
RSA	-.03	.51	-.13	.07
HR	-.001	.88	-.01	.009
Brooding*RSA	-.007	.58	-.03	.02
State Rumination (PMC)	.009	.32	-.01	.03
Brooding*State Rumination (PMC)	-.006	.007	-.01	-.002
RSA*State Rumination (PMC)	.02	.05	-.01	.03
Brooding*RSA*State Rumination (PMC)	-.01	.02	-.009	-.0007
Subjective stress (GMC)	.12	.01	.03	.21
Subjective stress (PMC)	.03	.003	.01	.05

Study 2

To examine reverse causality, we substituted the predictor and outcome variables.

Predicting brooding rumination

Effect	Estimate	p-value	Lower	Upper
Intercept	1.18	.80	-8.44	10.81
RSA	1.29	.006	.39	2.19
Chronic Interpersonal Stress	-.54	.25	-1.48	.40
RSA*Chronic Interpersonal Stress	.53	.21	-.32	1.39
Age	.02	.58	-.05	.08
Female	-.38	.73	-2.57	1.82
HR	.12	.04	.004	.24

Study 3

To examine reverse causality, we substituted the predictor and outcome variables.

	Mediation			95% Bootstrapped C.I. of Unstandardized Estimate	
Variables	b	S.E.	β	lower	upper
Outcome: Brooding					
Interpersonal Stress	-.117	.16	-.052	-.431	.197
Depression	.747**	.049	.73**	.651	.844
Group	-.076	.044	-.083	-.162	.009
Outcome: Reflection					
Interpersonal Stress	-.427	.222	-.189	-.836	.009
Depression	.523**	.067	.514**	.391	.655
Group	-.069	.054	-.075	-.174	.037
Outcome: Interpersonal Stress					
Depression	.15**	.044	.342**	.067	.241
Group	-.04	.034	-.097	-.107	.028
Correlation					
Brooding with Reflection	.269**	.039	.528**	.193	.344

Note. * $p \leq .05$; ** $p \leq .01$; Brooding, reflection, and depression are factor scores generated from the measurement model; Interpersonal stress is a latent variable; The model represents the path coefficients for the indirect effects model; Unstandardized coefficients (b), standard errors (S.E.), and standardized coefficients (β) are presented; 95% C.I. using 5000 bootstrapped samples; RSA = Respiratory sinus arrhythmia.

The indirect effect of depression on brooding rumination via interpersonal stress not significant (estimate = -.018, *SE* = .042, p = .484, bootstrapped 95% C.I. = -.069 to .032). The indirect effect of depression on reflective rumination via interpersonal stress was not significant (estimate = -.065, *SE* = .039, p = .09, bootstrapped 95% C.I. = -.144 to -.012).
